# Supplementary material for: Nutrient levels control root growth responses to high ambient temperature in plants
Source: Nat Commun. 2024 Jun 1;15:4689. doi: 10.1038/s41467-024-49180-6 (PMC11144241; doi:10.1038/s41467-024-49180-6)
Supplement: Supplementary file 1 — Supplementary Information [file 41467_2024_49180_MOESM1_ESM.pdf]

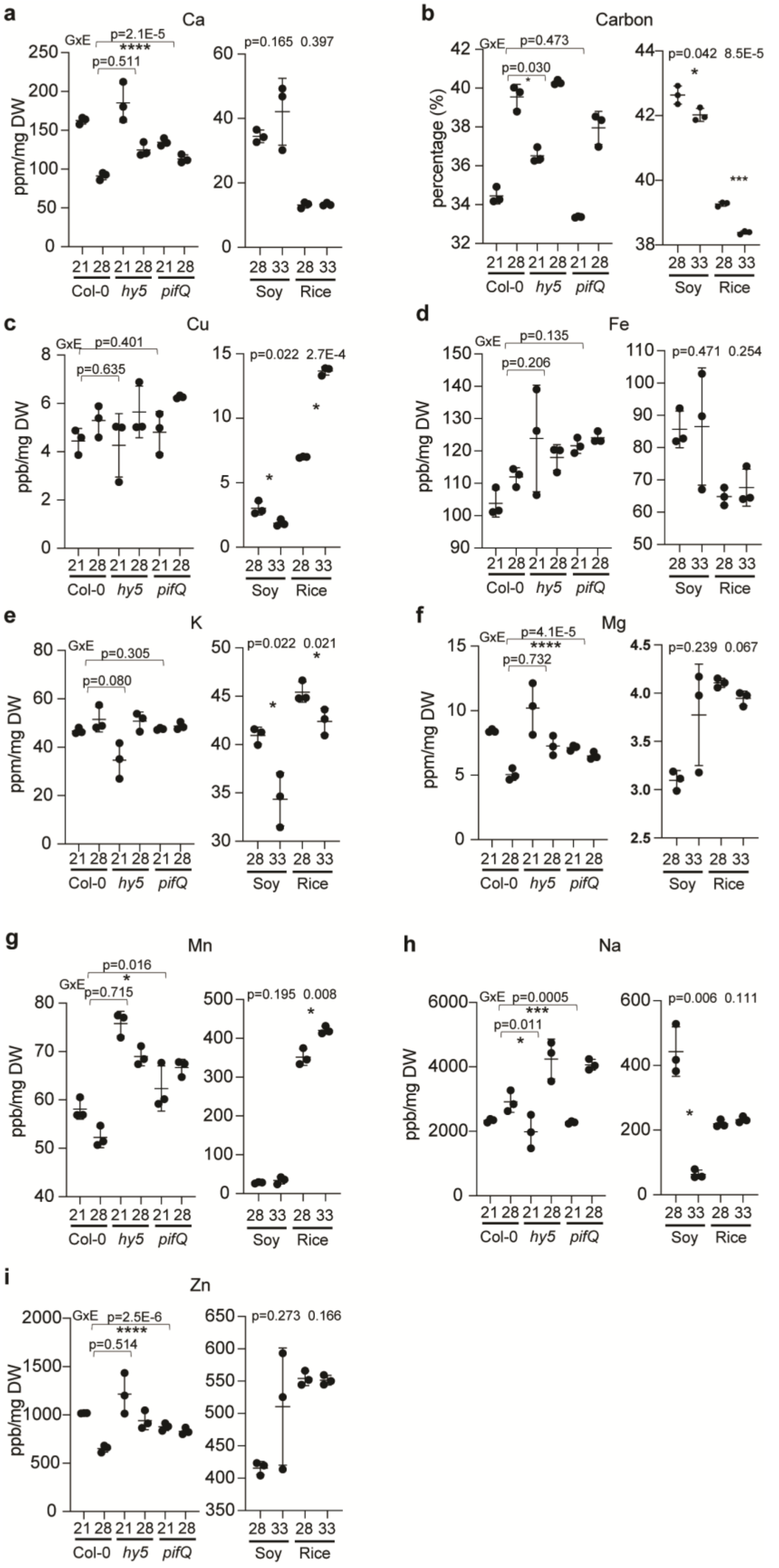

a
Ca
GxE p=2.1E-5
****
p=0.511
ppm/mg DW
p=0.165 0.397
21 28 21 28 21 28
Col-0 hy5 pifQ
28 33 28 33
Soy Rice
b
Carbon
GxE p=0.473
p=0.030
*
percentage (%)
p=0.042 * 8.5E-5
***
c
Cu
GxE p=0.401
p=0.635
ppb/mg DW
p=0.022 2.7E-4
*
*
d
Fe
GxE p=0.135
p=0.206
ppb/mg DW
p=0.471 0.254
e
K
GxE p=0.305
p=0.080
ppm/mg DW
p=0.022 0.021
*
*
f
Mg
GxE p=4.1E-5
****
p=0.732
ppm/mg DW
p=0.239 0.067
g
Mn
p=0.016
*
GxE p=0.715
ppb/mg DW
p=0.195 0.008
*
h
Na
GxE p=0.0005
***
p=0.011
*
ppb/mg DW
p=0.006 0.111
*
i
Zn
GxE p=2.5E-6
****
p=0.514
ppb/mg DW
p=0.273 0.166

Supplementary Figure 1. Additional nutrient analyses at higher temperatures in shoots of Arabidopsis, soybean, and rice.

a-i 9 additional nutrient analysis at higher temperature in three different species (Arabidopsis, soy, and rice) except nitrogen and phosphorus which are displayed in Fig.1 g-i. p-Values for the corresponding GxE interactions determined through ANOVA are shown on top of each graph. Asterisks indicate statistically significant difference either 2-way ANOVA; *p<0.05, **p<0.01, ***p<0.001, and ****p<0.0001. Average difference of each value is indicated. Shoot parts from 4-week-old plants from Arabidopsis, soybean, and rice plants were used for the nutrient analyses. Scatter dot plots indicate mean (horizontal line) and standard deviation (error bars).

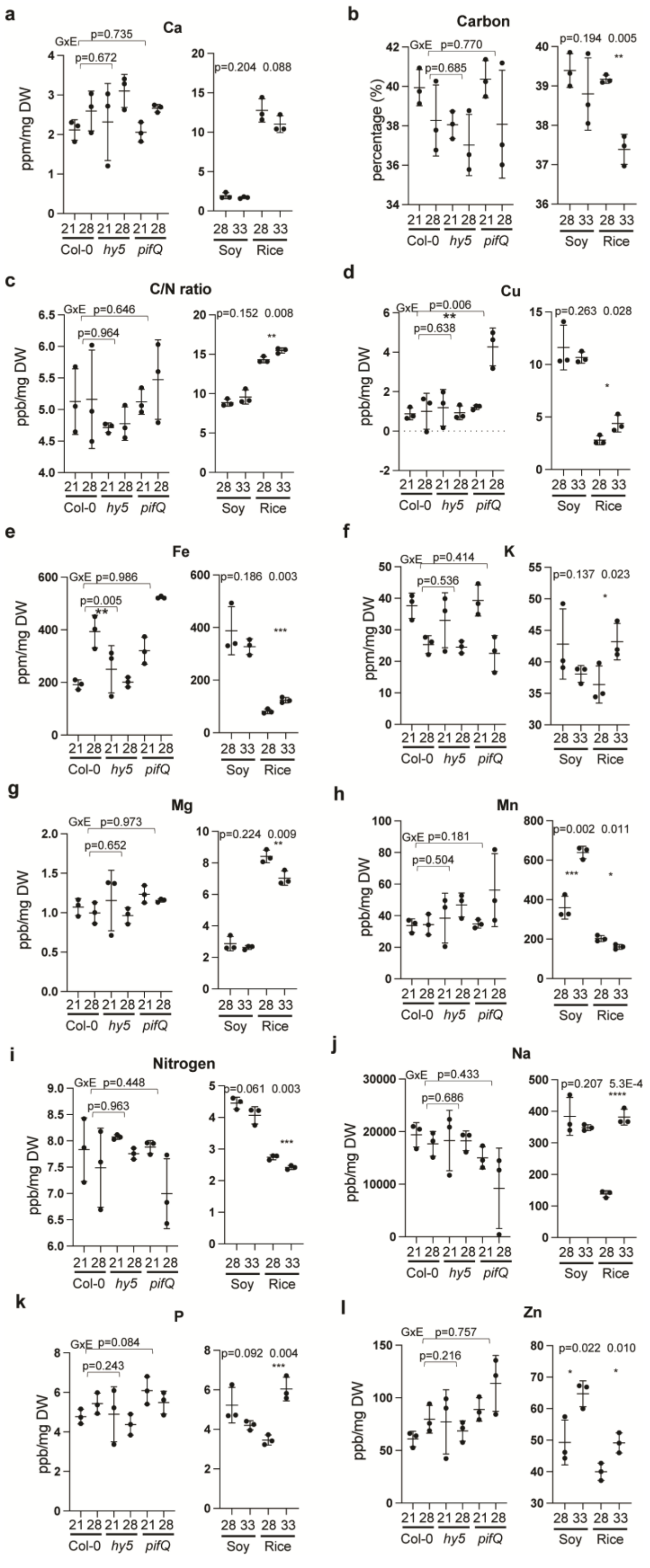
a
Ca
GxE p=0.735
p=0.672
ppm/mg DW
p=0.204 0.088
21 28 21 28 21 28
Col-0 hy5 pifQ
28 33 28 33
Soy Rice
b
Carbon
GxE p=0.770
p=0.685
percentage (%)
p=0.194 0.005
c
C/N ratio
GxE p=0.646
p=0.964
ppb/mg DW
p=0.152 0.008
d
Cu
GxE p=0.006
p=0.638
p=0.263 0.028
e
Fe
GxE p=0.986
p=0.005
p=0.186 0.003
f
K
GxE p=0.414
p=0.536
p=0.137 0.023
g
Mg
GxE p=0.973
p=0.652
p=0.224 0.009
h
Mn
GxE p=0.181
p=0.504
p=0.002 0.011
i
Nitrogen
GxE p=0.448
p=0.963
p=0.061 0.003
j
Na
GxE p=0.433
p=0.686
p=0.207 5.3E-4
k
P
GxE p=0.084
p=0.243
p=0.092 0.004
l
Zn
GxE p=0.757
p=0.216
p=0.022 0.010

Supplementary Figure 2. Nutrient analyses at higher temperatures using 9-day old seedling roots from Arabidopsis, soybean, and rice.

a-i 9 additional nutrient analysis at higher temperature in three different species (Arabidopsis, soy, and rice) except nitrogen and phosphorus which are displayed in Fig.1 g-i. p-Values for the corresponding GxE interactions determined through ANOVA are shown on top of each graph. Asterisks indicate statistically significant difference either 2-way ANOVA; *$p<0.05$, **$p<0.01$, ***$p<0.001$, and ****$p<0.0001$. Average difference of each value is indicated. Root parts from 9-day old plants from Arabidopsis, soybean, and rice plants were used for the nutrient analyses. Scatter dot plots indicate mean (horizontal line) and standard deviation (error bars).

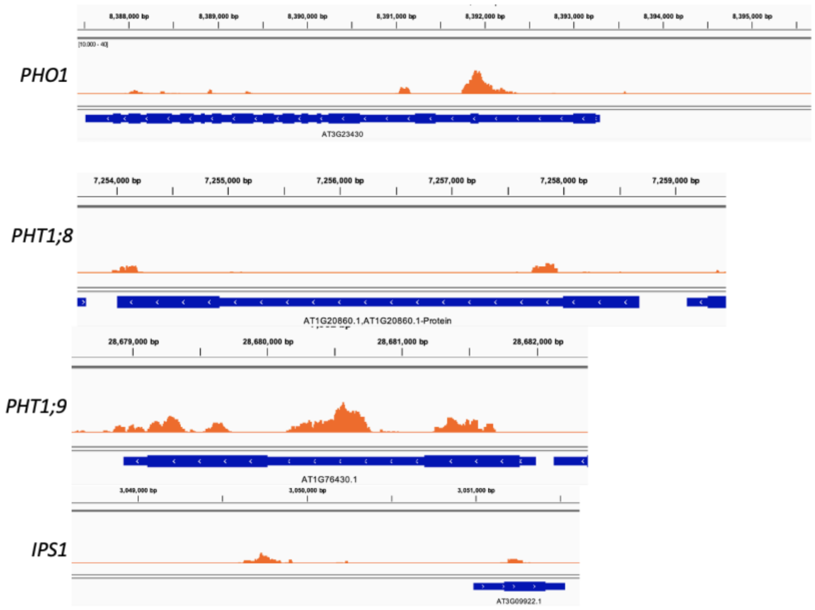

Supplementary Figure 3. Some P signaling genes are not the target of HY5 at high ambient temperature.

a IGV image of HY5 ChIP-seq data from Burko et al., (2020) of P signaling genes with transcription direction. In this study, we narrow-down the HY5 candidate binding sites with the value higher than 25.

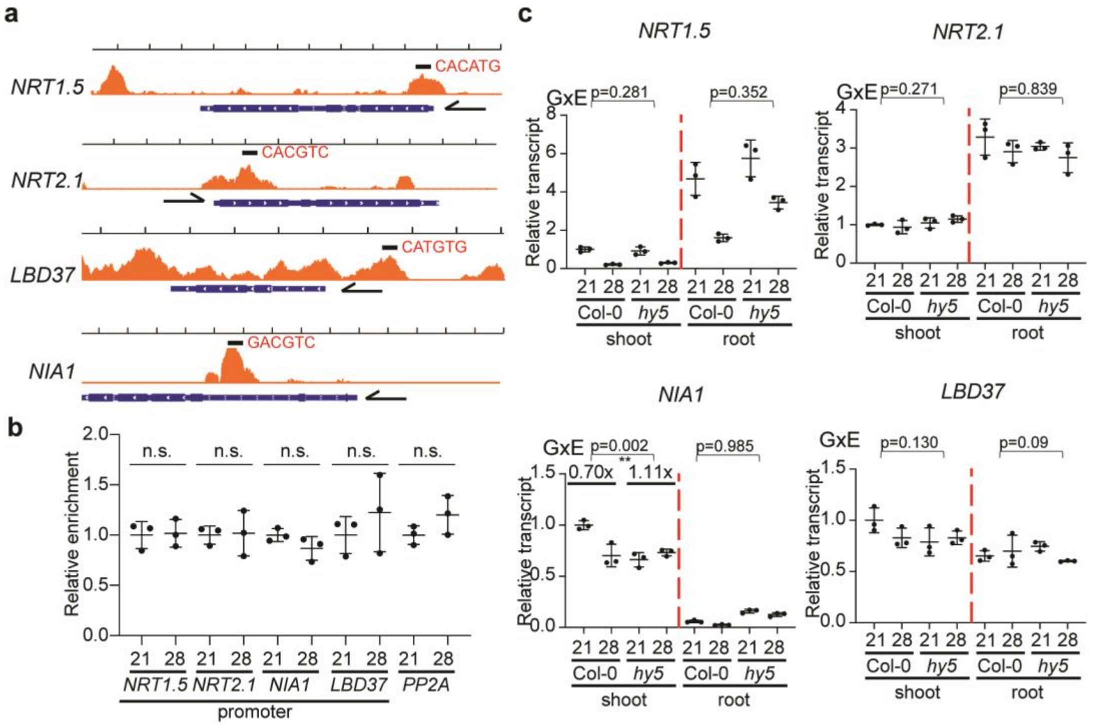

Supplementary Figure 4. Some N-P signaling genes are not the target of HY5 at high ambient temperature.

a IGV image of HY5 ChIP-seq data from Burko et al., (2020) of N-P signaling genes with transcription direction and binding motif. **b** Scatter dot plot of ChIP-qPCR results at normal and high ambient temperature using five different genes or promoter regions. **c** Scatter dot plot of qPCR results at normal and high ambient temperature using four different genes using Col-0 and *hy5-215* seedlings with shoot and root separate samples. Relative transcript level was normalized using *PP2A* as a control and to the expression levels in the shoot. p-Values for the corresponding GxE interactions determined through ANOVA are shown on top of each graph. Asterisks indicate statistically significant difference either 2-way ANOVA; *p<0.05, **p<0.01, ***p<0.001, and ****p<0.0001. Average difference of each value is indicated. Shoot and root samples were analyzed separately. Scatter dot plots indicate mean (horizontal line) and standard deviation (error bars)

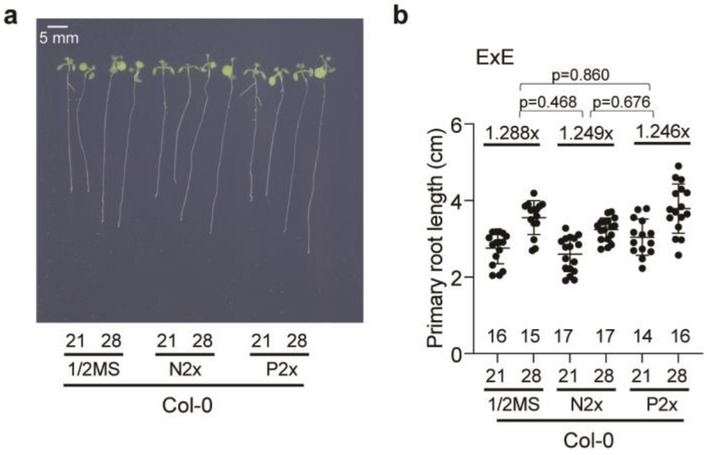

Supplementary Figure 5. Excessive Nitrogen and Phosphorus levels do not accelerate root thermomorphogenesis.

a Phenotypes of Col-0 in different media conditions at high ambient temperature. **b** Scatter dot plot of **a**. p-Values for the corresponding ExE interactions determined through ANOVA are shown on top of each graph. Asterisks indicate statistically significant difference using 2-way ANOVA. Average difference of each value is indicated. Average fold difference of each group is indicated in the top region of the plot. Scatter dot plots indicate mean (horizontal line) and standard deviation (error bars).

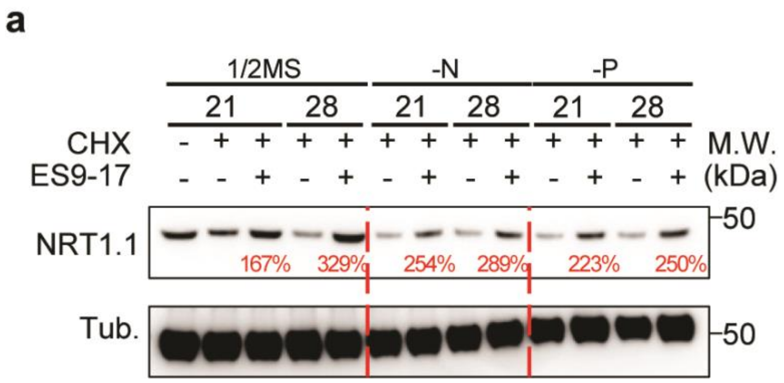

Supplementary Figure 6. Temporal NRT1.1 protein abundance is mostly determined by transcription level.

a Western blot analyses of NRT1.1 using the treatment of two inhibitors (CHX – Cycloheximide: protein synthesis inhibitor, ES9-17: clathrin-mediated endocytosis inhibitor) with temperature and nutrient deficient media conditions. Root samples were analyzed. 9 days old seedlings were pre-treated for 2 hours in the chemical containing solution and then transferred to either 21 or 28 for additional 4 hours. Concentration for CHX and ES9-17 was 50 uM and 30 uM respectively. Red number indicates the relative signal intensity in percentage comparing samples between the absence and presence of ES9-17 by divided by NRT1.1 signal to Tubulin.

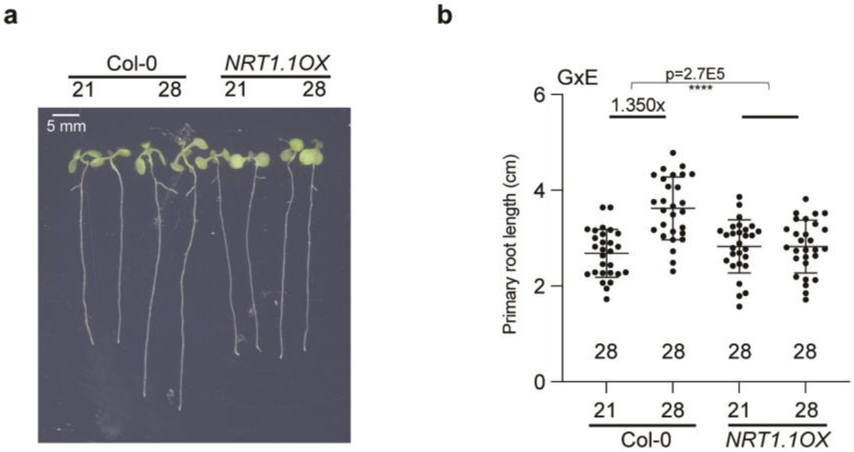

Supplementary Figure 7. Overexpression of NRT1.1 suppresses root thermomorphogenesis.
a-b Phenotypes of Col-0 in different media conditions at high ambient temperature. **b** Scatter dot plot of **a**. p-Values for the corresponding GxE interactions determined through ANOVA are shown on top of each graph. Asterisks indicate statistically significant difference using 2-way ANOVA. Average difference of each value is indicated. Average fold difference of each group is indicated in the top region of the plot. Scatter dot plots indicate mean (horizontal line) and standard deviation (error bars).

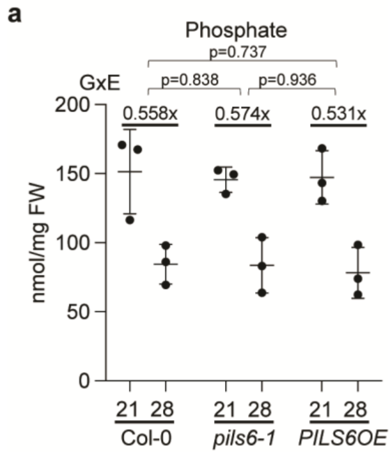

Supplementary Figure 8. PILS6 is not involved in P levels at high ambient temperature.
a Scatter dot plot of phosphate composition. Seeds of each genotype were grown on soil for 2 weeks at 21℃ and transferred into either 21℃ or 28℃ for additional 2 weeks. Then the leaves were used for the analyses. p-Values for the corresponding GxE interactions determined through ANOVA are shown on top of each graph. Asterisks indicate statistically significant difference using 2-way ANOVA. Average fold difference of each group is indicated in the top region of the plot. Scatter dot plots indicate mean (horizontal line) and standard deviation (error bars).

```
Glyma.16G092700      ------------------------------------------------------------     0
Glyma.03G081700      ------------------------------------------------------------     0
LOC_Os01g07880       ------------------------------------------------------------     0
Glyma.08G302500      MERSGGMVTGSHERNELVRVRHGSDSRSKPLKNLNGQSCQICGDTIGLTATGDVFVACHE     60
Glyma.18G117100      MERSGGMVTGSHERNELVRVRHGSDSGSKPLKNLNGQICQICGDTIGLTATGDLFVACHE     60
At5g11260            ------------------------------------------------------------     0
LOC_Os02g10860       ----------------------------------------MATTRA---SLTDPLLPSPA     17
LOC_Os06g39960       ------------------------------------------------------------     0


Glyma.16G092700      ------------------------------------------------------------     0
Glyma.03G081700      ------------------------------------------------------------     0
LOC_Os01g07880       ------------------------------------------------------------     0
Glyma.08G302500      CGFPLCHSCYEYELKHMSQSCPQCKTAFTSHQEGAEVEGDDDDEDDADDLDNEINYGQGN     120
Glyma.18G117100      CGFPLCHSCYEYELKNVSQSCPQCKTTFTSRQEGAEVEGDDDDEDDADDLDNGINYGQGN     120
At5g11260            ------------------------------------------------------------     0
LOC_Os02g10860       ARAPVKAKKLSWSMLHAS-----------SKDERRGQSGEA-------------------     47
LOC_Os06g39960       ------------------------------------------------------------     0


Glyma.16G092700      ---------------------MSLPRPSEGKAPSQLK-EGVAPAAAA-----ASSSSS--     31
Glyma.03G081700      ---------------------MSLPRPSEGKAPSQLK-EGVAPAAAE-----ASTSSS--     31
LOC_Os01g07880       -------------------------MAAQ--EQEQEKQQVKTSTTSSLPSSSERSSSSAP     33
Glyma.08G302500      SSKAGMLWEEDADLSSSSGHDSQIPNPHL--ANGQPM-SGEFPCATSDAQSMQTT--S--     173
Glyma.18G117100      NSKSGMLWEEDADLSSSSGHDSHIPNPHL--VNGQPM-SGEFPCATSDAQSMQTTSDP--     175
At5g11260            -------------------------------MQEQAT-SSLAASSL--PSSSERSSSSAP     26
LOC_Os02g10860       ----------EA--EASGGVHANPSSPAR--MQEQAT-SSRP-------SSSERSSSSGG     85
LOC_Os06g39960       -------------------------------MQRDHR-RSSPPAA---ASSSERSSDSAP     25
                                                       :                   :

Glyma.16G092700      ----------WNNRLHTFPPLSLHNKS-SKIEDSDEDMFTVPDVETTPVSVHSAATLQNS     80
Glyma.03G081700      ----------WNNRLNTFPPLSLHNKN-SKIEDSDEDMFTVPDVEATPINVHSAVTLQNS     80
LOC_Os01g07880       NNLKEG-----------------------GGVESDEEIRRVPEMGGGGGSASS-GAGADE     69
Glyma.08G302500      ------IGQSE--KVHSLSYAD----PKQPGPESDEEIRRVPEIGGESAGTSASQPDAGS     221
Glyma.18G117100      ------MGQSE--KVHSLPYAD----PKQPGPESDEEIRRVPEIGGESAGTSASRPDAGS     223
At5g11260            -HLEIKE-----------------------GIESDEEIRRVPEFGGEAVGKETSGRESGS     62
LOC_Os02g10860       HHMEIKEGKEAPLRSLLLPFLDFHFTVPLSGMESDEEIGRVPELGLEPGGASTSGRAAGG     145
LOC_Os06g39960       NTDDGKEG---------------------VDSDDEEIVGRVPEFGLALPGTSTSGRGSVR     64
                                                     :.:* :  **:.     .  :

Glyma.16G092700      NLTQ-----------RNVTDPQFQTGFPGKRRRGRNPADKEHRRLKRLLRNRVSAQQARE     129
Glyma.03G081700      NLNQ-----------RNVTDPQFQSGFPGKRRRGRNPADKEHRRLKRLLRNRVSAQQARE     129
LOC_Os01g07880       RQGKEDGKQQGGGGGGAAAAGGGQEQAPPARKRGRSAGDKEQNRLKRLLRNRVSAQQARE     129
Glyma.08G302500      NAGTER----------------VQGTGEGQKKRGRSPADKESKRLKRLLRNRVSAQQARE     265
Glyma.18G117100      NAGTER----------------AQGTGDSQKKRGRSPADKESKRLKRLLRNRVSAQQARE     267
At5g11260            ATGQER---------------TQATVGESQRKRGRTPAEKENKRLKRLLRNRVSAQQARE     107
LOC_Os02g10860       GGGGAE-------------RAQSSTAQASARRRGRSPADKEHKRLKRLLRNRVSAQQARE     192
LOC_Os06g39960       VAGDAAATAAGTSSSSPAAQAGVAGSSSSGRRRGRSPADKEHRRLKRLLRNRVSAQQARE     124
                                                   ::***. .:** .*****************

Glyma.16G092700      RKKVYVNDLESRAKELQDKNAILEERISTLINENTMLRKVLMNARPKTDDSIEQKQDQLS     189
Glyma.03G081700      RKKVYVNDLESRAKEMQDKNAILEERISTLINENTMLRKVLMNARPKNDDSIEQKQDQLS     189
LOC_Os01g07880       RKKAYMTELEAKAKDLELRNAELEQRVSTLQNENNTLRQILKNTTAHAGKRGGGGGGKGG     189
Glyma.08G302500      RKKAYLIDLETRVKDLEKKNSELKERLSTLQNENQMLRQILKNTTASRRGSNNGTNNAE-     324
Glyma.18G117100      RKKAYLIDLETRVKDLEKKNSELKERLSTLQNENQMLRQILKNTTASRRGSNSGTNNAE-     326
At5g11260            RKKAYLSELENRVKDLENKNSELEERLSTLQNENQMLRHVRFVDLTVDLTCNV-------     160
LOC_Os02g10860       RKKAYLNDLEVKVKDLEKKNSELEERFSTLQNENQMLRQILKNTTVSRRGPVLLKIPKSG     252
LOC_Os06g39960       RKKAYMSELEARVKDLERSNSELEERLSTLQNENQMLRQVLKNTTANRRGPDSSAGGDS-     183
                     ***.*: :** :.*:::  *: *::*.*** ***  **::

Glyma.16G092700      KS----------------------------------------------------------     191
Glyma.03G081700      KS----------------------------------------------------------     191
LOC_Os01g07880       DG-----GGGGKKH-----HFTKS------------------------------------     203
Glyma.08G302500      ------------------------------------------------------------     324
Glyma.18G117100      ------------------------------------------------------------     326
At5g11260            ---NAFAICFLIYHSTGLSHLRV-------------------------------------     180
LOC_Os02g10860       LREAAPAGCGGLREAEGDEKFVLNGFTAANLSFDGMATVTPNGLLMLTNGTNQLKGHAFF     312
LOC_Os06g39960       ------------------------------------------------------------     183


Glyma.16G092700      ------------------------------------------------       191
```

```
Glyma.03G081700      ------------------------------------------------      191
LOC_Os01g07880       ------------------------------------------------      203
Glyma.08G302500      ------------------------------------------------      324
Glyma.18G117100      ------------------------------------------------      326
At5g11260            ------------------------------------------------      180
LOC_Os02g10860       PALLQFHRTPNSMAMQSFSTAFVIGIISAFEDQGSGSPAAAGGSGRAA      360
LOC_Os06g39960       ------------------------------------------------      183
                     ***:* :   *.: : . :: * **  * *      : : :
```

Supplementary Figure 9. Protein alignment of HY5 homologs in Arabidopsis, soybean, and rice.

Multiple protein alignment of HY5 homologs were analyzed using Clustal Omega (1.2.4). Protein sequences are from 1 in Arabidopsis (At5g11260), 4 in soybean (Glyma. 01g210500, Glyma. 11g031500, Glyma. 17g139400, and Glyma. 05g056900), and 2 in rice (LOC_Os08g05910 and LOC_Os10g40600).

```
At1g12110           ------MSLPETKSDDILLDAWDFQGRPADRSKTGGWASAAMILCIEAVERLTTLGIGVN    54
Glyma.01G210500     ------MSSLPTTQGKPIPDASDYKGRPAERSKTGGWTASAMILGGEVMERLTTLGIAVN    54
Glyma.11G031500     ------MSNLPTTQGKAIPDASDYKGRPAERSKTGGWTASAMILGGEVMERLTTLGIAVN    54
Glyma.17G139400     ------MKTLPQTPGKTIPDACDYKGHPAERSKTGGWTAAAMILGVEACERLTTMGVAVN    54
Glyma.05G056900     ------MNTLPQTPGKTIPDACDYKGRPAERSKT----------GVEACERLTTMGVAVN    44
LOC_Os08g05910      MVGMLPETNAQAAAEEVLGDAWDYRGRPAARSRTGRWGAAAMILVAELNERLTTLGIAVN    60
LOC_Os10g40600      MAMVLPET---AAEGKALTDAWDYKGRPAGRAATGGWGCAAMILGAELFERMTTLGIAVN    57
                          .       . : ** *::*:** *: *            *  **:**:*:.**

At1g12110           LVTYLTGTMHLGNATAANTVTNFLGTSFMLCLLGGFIADTFLGRYLTIAIFAAIQA----    110
Glyma.01G210500     LVTYLTGTMHLGNAASANVVTNFLGTSFMLCLLGGFLADTFLGRYRTIAIFAAVQA----    110
Glyma.11G031500     LVTYLTGTMHLGNAASANVVTNFLGTSFMLCLLGGFLADTFLGRYRTIAIFAAVQA----    110
Glyma.17G139400     LVTYLTGTMHLGSANSANTVTNFMGTSFMLCLFGGFVADTFIGRYLTIAIFATVQAT---    111
Glyma.05G056900     LATYLTGTMHLGSANSANTVTNFMGTSLMLCLFGGFVADTFIGRYLTIAIFATVQATSQC    104
LOC_Os08g05910      LVTYLTATMHAGNAEAANVVTNFMGTSFMLCLLGGFVADSFLGRYLTIAIFTAVQA----    116
LOC_Os10g40600      LVPYMTGTMHLGNAAAANTVTNFIGTSFMLCLLGGFVADTYLGRYLTIAIFEAVQA----    113
                    *. *:*.*** *.* :**.****:***:****:***:**:::*** ***** ::**

At1g12110           -------------------------TGVSILTLSTIIPGLRPPRCNPT--TSSHCEQASG    143
Glyma.01G210500     -------------------------TGVTILTISTIIPSLHPPKCNGD--TVPPCVRANE    143
Glyma.11G031500     -------------------------TGVTILTISTIIPSLHPPKCNGD--TVPPCVRANE    143
Glyma.17G139400     --------------------------GVTILTISTIIPSLHPPKCIRD--ATRRCMPANN    143
Glyma.05G056900     KDIFLTHCNCIATAFVELYLYKICFHGVTILTISTIIPSLHPPKCIRD--ATRRCMSANN    162
LOC_Os08g05910      -------------------------SGVTILTISTAAPGLRPPAACAAG---SAACERATG    148
LOC_Os10g40600      -------------------------TGVMILTISTAAPGLRPPACGDPKGASAECVAADG    148
                                              ** ***:**  *.*:*  *         *  *

At1g12110           IQLTVLYLALYLTALGTGGVKASVSGFGSDQFDETEP-KERSKMTYFFNRFFFCINVGSL    202
Glyma.01G210500     KQLTALYLALYVTALGTGGLKSSVSGFGSDQFDDSDN-DEKKQMIKFFNWFYFFVSIGSL    202
Glyma.11G031500     KQLTVLYLALYVTALGTGGLKSSVSGFGSDQFDDSDD-DEKKQMIKFFNWFYFFVSIGSL    202
Glyma.17G139400     MQLMVLYIALYTTSLGIGGLKSSVSGFGTDQFDESDK-GEKKQMLKFFNWFVFFISLGTL    202
Glyma.05G056900     MQLMVLYIALYTTSLGIGGLKSSVSGFSTDQFDDSDK-GEKKQMLKFFNWFVFFISLGTL    221
LOC_Os08g05910      AQMGVLYLALYLTALGTGGLKSSVSGFGSDQFDESDS-GEKSQMMRFFNWFFFFISLGSL    207
LOC_Os10g40600      TQLGVLYLGLYLTALGTGGLKSSVSGFGSDQFDESDVDGERKKMMRFFNWFYFFVSLGAL    208
                      *: .**:.** *:** **:*:*****.:****:::   *:.:*  *** * * :.:*:*

At1g12110           LAVTVLVYVQDDVGRKWGYGICAFAIVLALSVFLAGTNRYRFKKLIGSPMTQVAAVIVAA    262
Glyma.01G210500     AATTVLVYVQDNIGRGWGYGICAGAIVVALLVFLSGTRKYRFKKRVGSPLTQFAEVFVAA    262
Glyma.11G031500     AATTVLVYVQDNIGRGWGYGICAGAIVVALLVFLSGTRKYRFKKLVGSPLTQFAEVFVAA    262
Glyma.17G139400     TAVTVLVYIQDHIGRYWGYGISVCAMLVALLVLLSGTRRYRYKRLVGSPLAQIAMVFVAA    262
Glyma.05G056900     TAVTVLVYIQDHIGRYWGYGISVCAMLVALLVLLSSTRRYRYKRLVGSPLTQIAMVFVAA    281
LOC_Os08g05910      LAVTVLVYVQDNLGRPWGYGACAAAIAAGLVVFLAGTRRYRFKKLVGSPLTQIAAVVVAA    267
LOC_Os10g40600      LAVTVLVYVQDNVGRRWGYGICAAGILAGLAVFLSGTRRYRFKKLVGSPLTQVAAVTAAA    268
                      *.*****:**.:** ****  .. .:   .* *:*:.*.:**:*: :***::*.* * .**

At1g12110           WRNRKLELPADPSYLYDVDDIIAA---------EGSMKGKQKLPHTEQFRSLDKAAIRDQ    313
Glyma.01G210500     LRKRNMELPSDSSLLFNDYD-----------------PKKQTLPHSKQFRFLDKAAIMDS    305
Glyma.11G031500     LRKRNMELPSDSSLLFNDYD-----------------PKKQTLPHSKQFRFLDKAAIMDS    305
Glyma.17G139400     WRKRHLEFPSDSSLLFNLDDVAD----------ETLRKNKQMLPHSKQFRFLDKAAIKDP    312
Glyma.05G056900     WRKRHLELPSDSSLLFNLDDVAD----------ESLRKNKQMLPHSKQFRFLDKAAIKDP    331
LOC_Os08g05910      WRKRRLELPSDPAMLYDIDVGKLAAAEVELAASSKKSKLKQRLPHTKQFRFLDHAAINDA    327
LOC_Os10g40600      WSKRSLPLPSDPDMLYDVDDAAAA---------GHDVKGKQRMPHSKECRFLDHAAIIDR    319
                      :* : :*:*   *::                     ** :**::: * **:*** *

At1g12110           EA-GVTSNVFNKWTLSTLTDVEEVKQIVRMLPIWATCILFWTVHAQLTTLSVAQSETLDR    372
Glyma.01G210500     SECGG--GMKRKWYLCNLTDVEEVKMVLRMLPIWATTIMFWTIHAQMTTFSVAQATTMDR    363
Glyma.11G031500     SECGG--GMKRKWYLCTLTDVEEVKMILRMLPIWATTIMFWTIHAQMTTFSVSQATTMDR    363
Glyma.17G139400     KTDGEEITMERKWYLSTLTDVEEVKMVQRMLPVWATTIMFWTVYAQMTTFSVQQATTMDR    372
Glyma.05G056900     KMDGEEITMQRNWYLSTLTDVEEVKMVQRILPVWATTIMFWTVYAQMTTFSVQQATTMDR    391
LOC_Os08g05910      PD-----GEQSKWTLATLTDVEEVKTVARMLPIWATTIMFWTVYAQMTTFSVSQATTMDR    382
LOC_Os10g40600      SAA-ESPATASKWRLCTRTDVEEVKQVVRMLPIWATTIMFWTIHAQMTTFAVAQAELMDR    378
```

```
                                :* *.. ******* : *:**:*** *:***::**:**::* *:  :**

At1g12110            SI--GSFEIPPASMAVFYVGGLLLTTAVYDRVAIRLCKKLFNYPHGLRPLQRIGLGLFFG     430
Glyma.01G210500      H-IGKTFQIPAASMTVFLIGTILLTVPFYDRFIVPVAKKVLKNPHGFTPLQRIGVGLVLS     422
Glyma.11G031500      H-IGKTFQMPAASMTVFLIGTILLTVPFYDRFIVPVAKKVLKNPHGFTPLQRIGVGLVLS     422
Glyma.17G139400      RIIGNSFQIPAASLTVFFVGSVLLTVPVYDRVITPIAKKLSHNPQGLTPLQRIGVGLVFS     432
Glyma.05G056900      R-IGNSFQIPAASLTVFFVGSVLLTVPIYDRVITPIAQKISHNPQGLTPLQRIGVGLVFS     450
LOC_Os08g05910       H-IGASFQIPAGSLTVFFVGSILLTVPIYDRLVVPVARRATGNPHGLTPLQRIGVGLVLS     441
LOC_Os10g40600       R-LAGGFLIPAGSLTVFLIASILLTVPFYDRLVVPVARRATANPHGLTPLQRVFVGLSLS     437
                           * :* .*::** :. :***. .***.   :.::    *:*: ****: :** :.

At1g12110            SMAMAVAALVELKRLRTAHAHGP---TVKTLPLGFYLLIPQYLIVGIGEALIYTGQLDFF     487
Glyma.01G210500      VISMVVGALIEIKRLRYAQSHGLVDKPEAKIPMTVFWLIPQNFIVGAGEAFMYMGQLNFF     482
Glyma.11G031500      VVSMVVGALIEIKRLRYAQSHGLVDKPEAKIPMTVFWLIPQNLFVGAGEAFMYMGQLDFF     482
Glyma.17G139400      ILAMVSAALIEIKRLRMARANGLAHKHNAVVPISVFWLVPQFFFVGSGEAFTYIGQLDFF     492
Glyma.05G056900      IFAMVSAALIEIKRLRMA----------------------QFFFVGSGEAFTYIGQLDFF     488
LOC_Os08g05910       IVAMVCAALTEVRRLRVARDARV--GGGEAVPMTVFWLIPQFLFVGAGEAFTYIGQLDFF     499
LOC_Os10g40600       IAGMAVAAAVERHRATA-----S--ASAAAAAPTVFLLMPQFLLVGAGEAFTYMGQLDFF     490
                       .*. .*  * :*                          * ::** ***: * ***:**

At1g12110            LRECPKGMKGMSTGLLLSTLALGFFFSSVLVTIVEKFTGKA---HPWIADDLNKGRLYNF     544
Glyma.01G210500      LRECPKGMKTMSTGLFLSTLSLGFFFSTLLVSIVNKMTAHG---RPWLADNLNQGRLYDF     539
Glyma.11G031500      LRECPKGMKTMSTGLFLSTLSLGFFFSTLLVSIVNKMTAHG---RPWLADNLNQGRLYDF     539
Glyma.17G139400      LRECPKGMKTMSTGLFLSTLSLGFFLSSLLVTLVHKATRHR---EPWLADNLNHGKLHYF     549
Glyma.05G056900      LRECPRGMKTMSTGLFLSTLSLGFFLSSLLVTLVHKATRHR---EPWLADNLNHGRLHHF     545
LOC_Os08g05910       LRECPKGMKTMSTGLFLSTLSLGFFVSSALVAAVHKLTGDR---HPWLADDLNKGQLHKF     556
LOC_Os10g40600       LRECPKGMKTMSTGLFLSTCAIGFFFSTLLVTIVHKVTGHGARGGGWLADNLDDGRLDYF     550
                     *****:*** *****:*** ::***.*: **: *.* * .      *:**:*:.*:*  *

At1g12110            YWLVAVLVALNFLIFLVFSKWYVYKEKRLAEVG---IELDDEPSIPMGH-     590
Glyma.01G210500      YWLLAILSAINVVLYLVCAKWYVYKEKRLADEG---IVLEETDDAAFHGH     586
Glyma.11G031500      YWLLAILSAINVVLYLVCAKWYVYKEKRLAEEC---IELEEADAAAFHGH     586
Glyma.17G139400      YWLLALLSGVNLVAYLFCAKGYVYKDKRLAEAG---IELEETDTASHA--     594
Glyma.05G056900      YWLLALLSGVNLVAYLFCAKGYVYKDKRLAEAG---IELEETDTACHA--     590
LOC_Os08g05910       YWLLAGVCLANLLVYLVAARWYKYKAGRAAAAGDGGVEMADAEPCLH*--     603
LOC_Os10g40600       YWLLAVISAINLVLFTVAARGYVYKEKRLADA---GIELADEETIAVGH*     596
                     ***:* :   *.: : . :: * **  * *      : : :
```

Supplementary Figure 10. Protein alignment of NRT1.1 homologs in Arabidopsis, soybean, and rice.

Multiple protein alignment of NRT1.1 homologs were analyzed using Clustal Omega (1.2.4). Protein sequences are from 1 in Arabidopsis (At1g12110), 4 in soybean (Glyma.01G210500, Glyma.11G031500, Glyma.17G139400, and Glyma.05G056900), and 2 in rice (LOC_Os08g05910 and LOC_Os10g40600).
