# Supplementary material for: Nutrient levels control root growth responses to high ambient temperature in plants
Source: Nat Commun. 2024 Jun 1;15:4689. doi: 10.1038/s41467-024-49180-6 (PMC11144241; doi:10.1038/s41467-024-49180-6)
Supplement: Supplementary file 3 — Description of Additional Supplementary Files [file 41467_2024_49180_MOESM3_ESM.pdf]

Description of Additional Supplementary Files:

Supplementary Data 1: Gene ontology analysis using RNAseq data from Gaillochet et al., (2020) and Lee et al., (2021), ChIPseq data from Burko et al., (2020).

Supplementary Data 2: Primers in this study.
